# Music of the 7Ts: Predicting and Decoding Multivoxel fMRI Responses with Acoustic, Schematic, and Categorical Music Features

**DOI:** 10.3389/fpsyg.2017.01179

**Published:** 2017-07-14

**Authors:** Michael A. Casey

**Affiliations:** Bregman Music and Audio Lab, Computer Science and Music Departments, Dartmouth College Hanover, NH, United States

**Keywords:** multivariate, fMRI, naturalistic, music-informatics, stimulus-encoding, genre, melody, harmony

## Abstract

Underlying the experience of listening to music are parallel streams of auditory, categorical, and schematic qualia, whose representations and cortical organization remain largely unresolved. We collected high-field (7T) fMRI data in a music listening task, and analyzed the data using multivariate decoding and stimulus-encoding models. Twenty subjects participated in the experiment, which measured BOLD responses evoked by naturalistic listening to twenty-five music clips from five genres. Our first analysis applied machine classification to the multivoxel patterns that were evoked in temporal cortex. Results yielded above-chance levels for both stimulus identification and genre classification–cross-validated by holding out data from multiple of the stimuli during model training and then testing decoding performance on the held-out data. Genre model misclassifications were significantly correlated with those in a corresponding behavioral music categorization task, supporting the hypothesis that geometric properties of multivoxel pattern spaces underlie observed musical behavior. A second analysis employed a spherical searchlight regression analysis which predicted multivoxel pattern responses to music features representing melody and harmony across a large area of cortex. The resulting prediction-accuracy maps yielded significant clusters in the temporal, frontal, parietal, and occipital lobes, as well as in the parahippocampal gyrus and the cerebellum. These maps provide evidence in support of our hypothesis that geometric properties of music cognition are neurally encoded as multivoxel representational spaces. The maps also reveal a cortical topography that differentially encodes categorical and absolute-pitch information in distributed and overlapping networks, with smaller specialized regions that encode tonal music information in relative-pitch representations.

## 1. Introduction

Humans possess an effortless proclivity to enjoy musical experiences in a wide variety of styles and acoustic configurations. Being moved by, or moving to music requires mental processing that is sensitive to specific auditory and schematic information–the precise features of which, as well as their cortical organization, are yet to be properly understood. Substantial progress has been made in eliciting the tuning response of groups of voxels to acoustic features in primary auditory areas (Aertsen and Johannesma, 1981; Eggermont et al., 1981; Cariani and Delgutte, 1996; Bendor and Wang, 2005; McDermott and Oxenham, 2008). However, far less is known about responses to categorical and schematic music features–such as genre categories and pitch classes–and about music representations that are encoded outside of primary auditory areas. We address the gap in understanding the fundamental neural codes underlying music cognition by combining methods from three research fields: (i) music cognition, (ii) music information retrieval, and (iii) multivoxel pattern analysis applied to high-field functional magnetic resonance imaging (fMRI).

### 1.0.1. Multidimensional representations in music cognition and music informatics

Results of music cognition research show that multidimensional geometries are implicated in the encoding of musical attributes and in processes of anticipation and reward during music perception. Examples of such geometries include the pitch spiral, torus, and tonnetz models of tonal pitch cognition (Shepard, 1964; Krumhansl, 1990; Tymoczko, 2012), and simplex models of categorical rhythm perception (Honing, 2012). These studies demonstrated that common behavioral responses to music are predicted by models employing statistical learning within multidimensional geometric spaces.

Likewise, music information retrieval systems learn embeddings of musical features in multidimensional spaces, the geometric properties of which are used to successfully predict behavior such as music categorization and musical preferences (Bartsch and Wakefield, 2001; Tzanetakis et al., 2002). Such representations are widely adopted for products and services relating to music consumption (Casey et al., 2008). Hence, a portion of the information in music is inherently geometric, and the properties of such geometries correspond with human behavior.

### 1.1. Prior work

#### 1.1.1. Voxel encoding and decoding models

Direct testing of hypotheses about cognitive representations of music and their topographies can be achieved with stimulus-model-based encoding and decoding. Janata et al. (2002) used the geometric pitch-torus model described by Krumhansl (1990), which preserves pitch-distance relationships as perceived by listeners. In their fMRI study, moment-to-moment pitch information of the stimulus–a clarinet melody cycling through all keys–was projected onto a pitch torus using an artificial neural network model (self-organizing map), and the model outputs were used as inputs to a regression model with fMRI voxel responses as the dependent variables. Clusters of significant model predictions were found in pre-frontal cortex, predominantly in rostral and ventral reaches of superior frontal gyrus (SFG). Also utilizing schematic stimulus-model-based encoding, Foster and Zatorre (2010) studied absolute- and relative-pitch representations in a melody-transposition memory task. Their results implicated the intraparietal sulcus (IPS) in comparing two differently transposed melodies.

Expanding the scope of topographical mapping of music features, Alluri et al. (2012) used 25 acoustic features automatically extracted from a single naturalistic musical work–a tango of 8 min duration–to investigate voxel responses to timbral, rhythmic, and tonal features voxel-wise for large cortical and subcortical volumes. Results showed anatomically distinct responses between the three feature groups. Timbral features were implicated in HG, STG, rolandic operculum (ROL), supramarginal gyrus (SMG), superior temporal pole (STP), and the cerebellum; rhythmic and tonal features were found in STG, inferior temporal gyrus (ITG), precuneus, and several subcortical limbic areas–including the left hemispheric amygdala, hippocampus and putamen, mid-cingulate gyrus, supplementary motor area, and the insula. In a further study, they were able to predict voxel responses in bilateral auditory cortex to two music medleys (Alluri et al., 2013), showing significant accuracy of voxel response predictions for auditory, limbic, motor, somatosensory, and frontal areas. In a related work, Toiviainen et al. (2014) demonstrated decoding of acoustic features, predicting the stimulus feature from the voxel response. They found contributions from STG, HG, ROL, and cerebellum contributed to the decoding of timbral features. Bilateral STG, right HG, and hippocampus were significant for rhythmic features. Tonal features, however, were not predicted above chance levels in their study, leaving open the question of whether multivoxel patterns are required to accurately decode neural representations of tonality.

#### 1.1.2. Multivoxel pattern analysis

Multivariate pattern analysis (MVPA) treats voxels as the dimensions of continuously-valued feature spaces, such that stimulus-evoked activations are distributed and overlapping between distinct conditions (Haxby et al., 2001; Kriegeskorte et al., 2006; Kriegeskorte, 2011; Stelzer et al., 2013). MVPA models of information representation may recruit the same set of voxels in two or more stimulus conditions with different response levels in each (Haxby et al., 2014).

Applying multivoxel pattern analysis to music, Casey et al. (2012) showed that timbral features based on cepstral coefficients most accurately predicted voxel patterns in primary and secondary auditory areas: Heschl's gyrus (HG), superior temporal gyrus (STG), and superior temporal sulcus (STS). Guntupalli (2013) repeated the experiment of Casey et al. (2012), and additionally performed whole-brain hyperalignment to create between-subject models of stimulus encoding and reconstruction for spectral and timbral acoustic features. Lee et al. (2011) also used voxel-based decoding to classify melodic contour of ascending and descending major and minor scales.

### 1.2. Hypothesis

Our central hypothesis is that distinct musical attributes are neurally encoded as multivoxel representational spaces. The dimensions of these spaces are individual voxel responses that, when analyzed together in a region, yield properties corresponding to musical behaviors. As such, we would expect machine learning models to statistically infer and generalize the patterns in these encodings, thus yielding accurate decoding of music information from multivoxel patterns elicited by novel stimuli (decoding models) and accurate predictions of multivoxel patterns for features of novel stimuli (stimulus-model-based encoding).

We also hypothesize that, for naturalistic music listening, multivoxel representational spaces will span the hierarchy of music information from the most general–such as musical style and genre–to the specific–such as melody encoded as relative pitch classes. We further hypothesize that distinct musical features will be differentially encoded across regions where music information is processed, including temporal, pre-frontal, frontal, parietal, occipital, hippocampal, and cerebellar regions, as implied by the prior research outlined above.

We focus our investigation of multivoxel representations on different levels of musical representation: high-level categorical features (5-category music genre), schematic melody features in absolute- and relative-pitch representations, and harmony features encoded by acoustic pitch-class profiles, also called chromagrams. The remainder of the paper proceeds as follows: Section 2 describes the stimuli, experimental procedure, and fMRI data collection and processing; Section 2.4 details the data analysis methods; results are presented in Section 3 followed by discussion of the results and their implication for music cognition in Section 4; and we conclude in Section 5 by outlining directions for our future research.

## 2. Materials and methods

### 2.1. Participants

We used the public OpenFMRI dataset published in Hanke et al. (2014) and Hanke et al. (2015). The subject pool consisted of 20 right-handed participants (mean age: 26.6 years, 12 male) who responded to a bulletin calling for volunteers for the study. All participants were native German speakers, and they all reported to have normal hearing without permanent or current temporary impairments and with no known history of neurological disorders. Each participant filled out a questionnaire, detailing basic demographic information, as well as music preference, proficiency and education. As detailed in Hanke et al. (2014) “Participants were fully instructed about the nature of the study, and gave their informed consent for participation in the study as well as for publicly sharing all obtained data in anonymized form. They were paid 100 EUR for their participation. The study was approved by the ethics committee of the Otto-von-Guericke-University of Magdeburg, Germany” (approval reference 37/13).

### 2.2. Stimuli and procedure

Stimuli used in this study were identical to those used in three previous studies: Casey et al. (2012), Guntupalli (2013), and Hanke et al. (2015), and are made publicly available in the OpenFMRI *Study Forrest* dataset (Hanke et al., 2014). Twenty five stereo, high-quality naturalistic music stimuli (6 s duration; 44.1 kHz sampling rate) were acquired, with five stimuli in each of five different music genres: (1) Ambient, (2) Country (3) Heavy Metal, (4) RocknRoll, and (5) Symphonic, see Table 1. Each stimulus consisted of a six-second excerpt from the middle of a distinct music recording captured from a high-quality Internet streaming service that was seeded by a representative artist for each genre. Clips were manually aligned to the nearest metrical down beat, and they were energy balanced so that the root-mean-square power value was equal across clips. A 50 ms quarter-sine ramp was applied at the start and end of each excerpt to suppress transients. The most prominent differences between the music clips were the presence or absence of vocals and percussion.

**Table 1:** List of stimuli used in experiments showing details of music genres, (seed artist), title, artist, and musical key for each clip.

**Style/(Seed artist)**	**Title**	**Artist**	**Key (Clip)**
Ambient			
(Brian Eno)	A Clearing	Brian Eno	F
	Theme from “Creation”	Brian Eno	C
	Old Land	Eno Moebius Roedelius	C
	Horizons Lointains	Galerie Stratique	Cm
	IO - Moon of Jupiter	Anugarma	B
Country			
(Waylon Jennings)	Are You Sure…?	Waylon Jennings	C
	Me and Paul	Willie Nelson	A
	Pancho and Lefty	Merle Haggard	D
	Whiskey Bent and Hell Bound	Hank Williams Jr.	G
	Welfare Line	Willie Nelson	D
Heavy Metal			
(Ozzy Osbourne)	Fire in the Sky	Ozzy Osbourne	D♭
	You've Got Another Thing Coming	Judas Priest	F♯
	Of Wolf & Man	Metallica	E
	You Shook Me All Night Long	AC-DC	G
	Rock You Like A Hurricane	Scorpions	Em
Rock & Roll			
(Eddie Cochran)	Jailhouse Rock	Elvis Presley	E♭
	Shake Rattle and Roll	Bill Haley	F
	Bama Lama Bama Loo	Little Richard	F
	Come On Let's Go	Ritchie Valens	A
	Money Honey	Eddie Cochran	E
Symphonic			
(Beethoven)	Symphony No. 9 Mvt. 2	Beethoven	F
	Symphony No. 4 Mvt. 4	Tchaikovsky	B♭m
	Symphony No. 2 Mvt. 4	Sibelius	D
	Symphony No. 5 Mvt. 1	Schubert	F
	Symphony No. 6 Mvt. 1	Beethoven	F

*All clips were 6 s duration, acquired from 44.1 kHz stereo 192 kbps streams*.

Procedures and stimulation setup were as previously reported in Hanke et al. (2014). Participants listened to the audio using custom-built in-ear headphones. After an initial sound calibration, eight scanning runs were performed with each run started by the participant with a key-press ready signal. There were 25 trials, with five different stimuli for each of the five genres per run. Stimulus genre ordering was 2nd-order sequence counter-balanced using De Bruijn cycles. Scanning was continuous, with a delay of 4 s, 6 s, or 8 s between trials. The order of delays was also randomized within each run. Five times per run, once per genre, participants were presented with a question asking for a Yes/No response to a particular feature of the stimulus: e.g., “Was there a female singer?” “Did the song have a happy melody?” The questions were designed to keep subjects' attention on the listening task. Participants were given inter-run breaks, with most resting for under a minute between runs. Stimulus presentation and response logging were implemented using PsychoPy running on a computer with the (Neuro)Debian operating system.

#### 2.2.1. Schematic and acoustic features extraction

**Figure 1:**
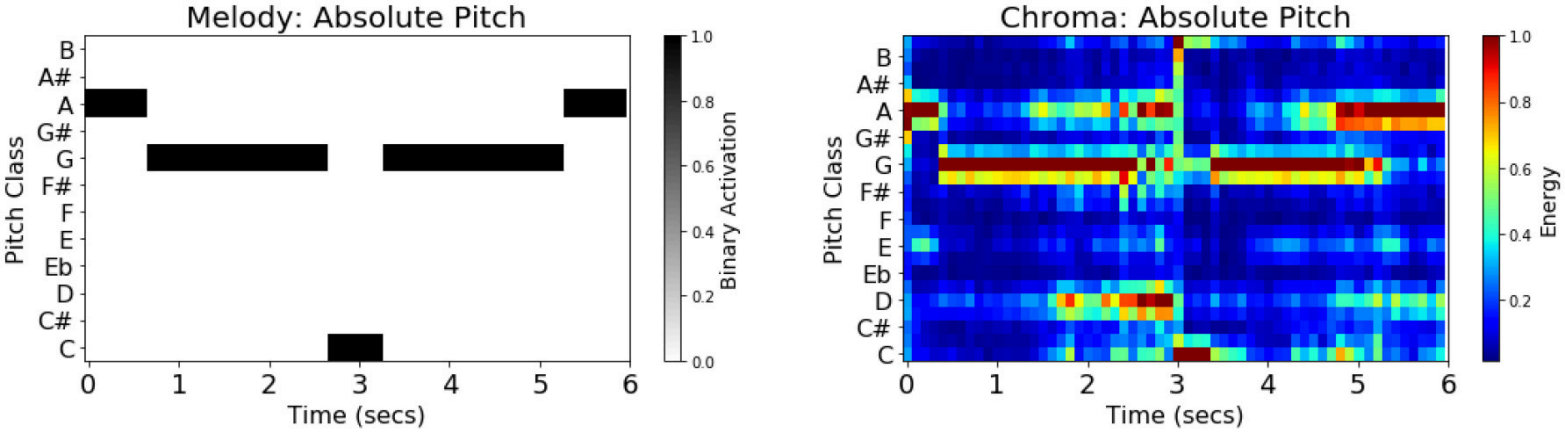
**(Left)** Schematic melody features (absolute pitch) for stimulus Ambient002. The binary-valued indicator matrices were obtained by three independent human expert transcriptions, followed by machine encoding to absolute time-scale and relative-pitch representation. **(Right)** Audio chromagram features (absolute pitch) for the same stimulus automatically extracted using the *Essentia* audio feature extraction toolkit in Python. Visible in the diagram is the trait that chromagram features are polyphonic, encoding all pitches present in the music clip, such as those corresponding to bass and chords, in addition to the melody.

In addition to genre labels, the following musical features were extracted from each stimulus: melody schema (absolute pitch), melody schema (relative pitch), and acoustic chromagram features (absolute pitch). The melodies for each of the twenty-five 6-second stimuli were annotated manually by two music undergraduate students and one music graduate student, using the ABC symbolic music standard (Oppenheim, 2010) with discreet pitch-classes aligned to a tempo-invariant metrical grid quantized by 16th-notes. The three sets of annotations were subsequently compared to achieve maximal agreement. These human transcriptions were automatically converted to schematic observation matrices consisting of 12-dimensional absolute pitch-class binary indicator vectors both in the original key (absolute pitch), and transposed to the key of C (relative pitch). Annotations were automatically re-sampled from tempo-normalized 16th-note metrical locations to an absolute time-scale of regular 0.1 s sample intervals, using stimulus tempo information, yielding a 60 × 12 observation matrix per stimulus. Figure 1 shows the absolute-pitch melody binary indicator matrix and the corresponding chromagram feature matrix for “Theme from ‘Creation’” by Brian Eno, which is the second stimulus in the Ambient category.

Schematic features are invariant to non pitch-class variation in the stimulus, such as loudness fluctuations, timbre, frequency content, articulation, and spatial information. To test whether such variation would be a confounding factor, we also extracted acoustic chromagram features–continuous-valued energies of equal-temperament pitch-class profiles extracted via the Essentia audio MIR toolkit (Bogdanov et al., 2013). Among the numerous differences between acoustic chromagrams and binary-chord schema are the presence of continuous energy values, amplitude modulation (due to loudness and dynamics), spectral envelope modulation (due to timbre), energy (from melody and bass notes and their harmonics), mis-aligned frequency channels (tuning), harmonic energy, room acoustics, and additive noise–to enumerate just a few differences. All features, schematic and acoustic, were further processed by singular-value decomposition, preserving the coefficients that explained at least 95% of the feature variance across the training stimulus set. As with the EPI features, feature matrices were flattened into vectors by stacking the 60 observation vectors (60 × 0.1 s samples) for each stimulus, thereby preserving their temporal sequence information, prior to subsequent data analysis.

### 2.3. fMRI data acquisition and pre-processing

The high-resolution 7-Tesla fMRI data was previously released via the *OpenFMRI* initiative (Hanke et al., 2015); the stimuli and experiment design used in the music perception phase of the data release (scanning session III) reproduce the original 3T experiment of Casey et al. (2012). To our knowledge, the current study is the first feature-based analysis of the music representational spaces revealed by the published high-resolution data set.

Functional MRI data was recorded during auditory stimulation. Anatomical T1-weighted scans were performed at 3 Tesla, and T2^*^-weighted functional scans were performed at 7 Tesla for slabs with partial brain coverage (MNI152 *z* ≈ −30 mm…40 mm). Subjects were given the cognitive task of listening attentively to the twenty five music clips in five genres, as shown in Table 1, and answering a dual-choice question, e.g., “did the clip have a happy melody?” Subjects responded “yes” or “no” to these questions via a response button box. These questions helped to ensure that subjects attended to the music across trials. Data from these catch trials were discarded from the analyses. The process was repeated eight times for each participant, using a unique quasi-randomized second-order balanced stimulus sequence for each subject and for each of the eight acquisition runs. Data consisted of 153 volumes per run, with a repetition time (TR) of 2.0 s each volume. Following is a summary of details of scanning, motion correction, and distortion processing as described in Hanke et al. (2014). T2^*^-weighted echo-planar images were acquired during stimulation using a 7-Tesla Siemens MAGNETOM magnetic resonance scanner. Thirty six axial slices (thickness 1.4 mm, 1.4 × 1.4 mm in-plane resolution, 224 mm field-of-view (FoV), anterior-to-posterior phase encoding direction) with a 10% inter-slice gap were recorded in ascending order. This configuration was chosen to achieve a balance between spatial resolution, volume coverage and volume acquisition time. Slices were oriented to include the ventral portions of frontal and occipital cortex while minimizing intersection with the eyeballs. The field-of-view was centered on the approximate location of Heschl's gyrus. Head-movement correction utilized reference scans at the start of the recording session and was performed on-line within the scanner in conjunction with a high-field distortion correction procedure.

EPI images were co-registered to a common group template using FSL's FLIRT, MCFLIRT, and FNIRT software. A group-specific template volume for EPI images was derived in order to aid anatomical alignment across brains. Subject's functional images were aligned to their respective reference-scan images, acquired at the start of the session, via a rigid body transformation using MCFLIRT. Each subject's reference-aligned images were averaged to create a template image for each brain. Subsequently, all subjects' template images were aligned by means of an affine transformation using FLIRT. The affine transformation was determined using the subject's brain with the least root mean square difference to the average image across all brains prior to alignment. The resulting average template volume was masked to produce the maximal intersection of individual brains to create the group EPI template volume (Hanke et al., 2014).

EPI data were then projected to voxel features using a per-voxel General Linear Model (GLM) for each stimulus in each run. The GLM was fitted for the EPI voxel time series in each run using the PyMVPA software framework (Hanke et al., 2009). The model fitting algorithm used the event-related design matrix (e.g., 3 × 2 s TRs per 6-s stimulus condition) with a double-gamma hemodynamic response function (HRF) regressor.

### 2.4. Analysis

#### 2.4.1. Analysis 1: mulivoxel classification by song and by music genre

Within-subject classifiers were trained on two tasks: song (stimulus) classification and genre (category) classification. After feature selection using a held-out portion of the dataset, song classifiers were cross-validated by run, and genre classifiers were cross-validated by stimulus–with category balancing achieved by holding out all runs of one stimulus from each of the five categories per cross-validation fold. We used linear-kernel support vector machines (SVM) with margin-parameter, *C*, scaled according to the norm of the data.

##### 2.4.1.1. Region of interest specification

Three bilateral regions in temporal cortex were selected from the Harvard-Oxford Cortical Structural Atlas, using FSLVIEW's Atlas Tools, and then warped to each subject's brain via the common group template. Regions of interest (ROIs) were selected spanning primary and secondary auditory cortex due to their implication in prior music classification studies (Casey et al., 2012; Guntupalli, 2013); these were: Heschl's gyrus (HG), anterior superior temporal gyrus (aSTG), and posterior superior temporal gyrus (pSTG).

To reduce the impact of noisy voxels on classifier performance, sensitivity-based feature selection retained only the top 5,000 voxels in each ROI. One-way analysis of variance (ANOVA), with individual stimulus factors, was applied followed by sensitivity-based feature selection, keeping only 5,000 voxels with the highest F-scores. To address possible circularity bias between feature selection and model training and testing, e.g., see Kriegeskorte et al. (2009), runs 1 and 4 were held out for feature selection and the remaining six runs were used for model training and cross-validation. Z-score mapping of the fMRI data was folded into the cross validation. Analysis scripts were implemented in Python 2.7.12 using the Anaconda distribution and the PyMVPA 2.6.0 framework (Hanke et al., 2009).

#### 2.4.2. Analysis 2: stimulus-encoding model searchlight

The anatomical distribution of cognitive music representations was analyzed using a searchlight algorithm (Kriegeskorte et al., 2006; Haxby et al., 2014). This procedure yielded an anatomical map of stimulus-model-based prediction accuracies in spherical subsets (“searchlights”) centered on every voxel; the map value for each voxel thus derives from the information present in each searchlight volume, and not each voxel individually. Stimulus encoding models were trained and tested for each of ≈6, 250 searchlight volumes–varied by subject anatomy–over a large volume of cortex–all Harvard-Oxford Cortical Atlas regions within the field of view, including pre-frontal, frontal, parietal, occipital, para-hippocampal, and cerebellar regions–using ridge regression with music stimulus features as input variables and voxel pattern responses as the dependent variables. The models were used to predict the voxel pattern response vector for new stimuli on the basis of their extracted musical features. Ridge regression was chosen due to its use of Tikhonov regularization, to counter possible deleterious effects of overdetermined models and other numerical instabilities.

A sphere-radius of 3 voxels was used and the accuracy of the predictions was defined as the correlation-error probability (1−*p*) between model predictions and voxels in each searchlight volume. The correlation-error probability yielded a measure in the range [0…1], with perfect predictions scoring 1. The searchlight creates ROIs by exhaustive subset selection, therefore we did not need to hold runs out for feature selection as we did in Analysis 1. For testing on novel data, balanced cross-validation held out all 8 runs of a randomly-selected stimulus in each of the five genre categories. Cross-validation was repeated 10 times in each searchlight, yielding 5-stimuli × 8 runs × 10 repetitions = 400 tests per searchlight, which were averaged to give a single correlation-error probability score per searchlight. Due to the large computational demand of searchlight analysis, we used randomized scattering by 3 voxels, and averaged results over the multiple cross-validation folds, which sped-up the computation by a factor of 27 relative to a searchlight sphere spacing of 1 voxel. The searchlight analysis, and permutation computations for bootstrapping the null distribution, took approximately 15,000 h of CPU time using scattering, so the speed-up factor was critical to the computational feasibility of the results. The searchlight with radius 3 voxels yielded spheres containing a maximum of 123 voxels for each center location. Following the methods of Stelzer et al. (2013), group-level statistical evaluation of the searchlight analysis was implemented using 100, 000 bootstrap samples drawn pair-wise by subjects from 100 randomized-target null models in each searchlight, and then estimating a voxel-wise threshold with probability *p* < 0.001 with respect to the bootstrap null distribution.

## 3. Results

### 3.1. Analysis 1

**Figure 2:**
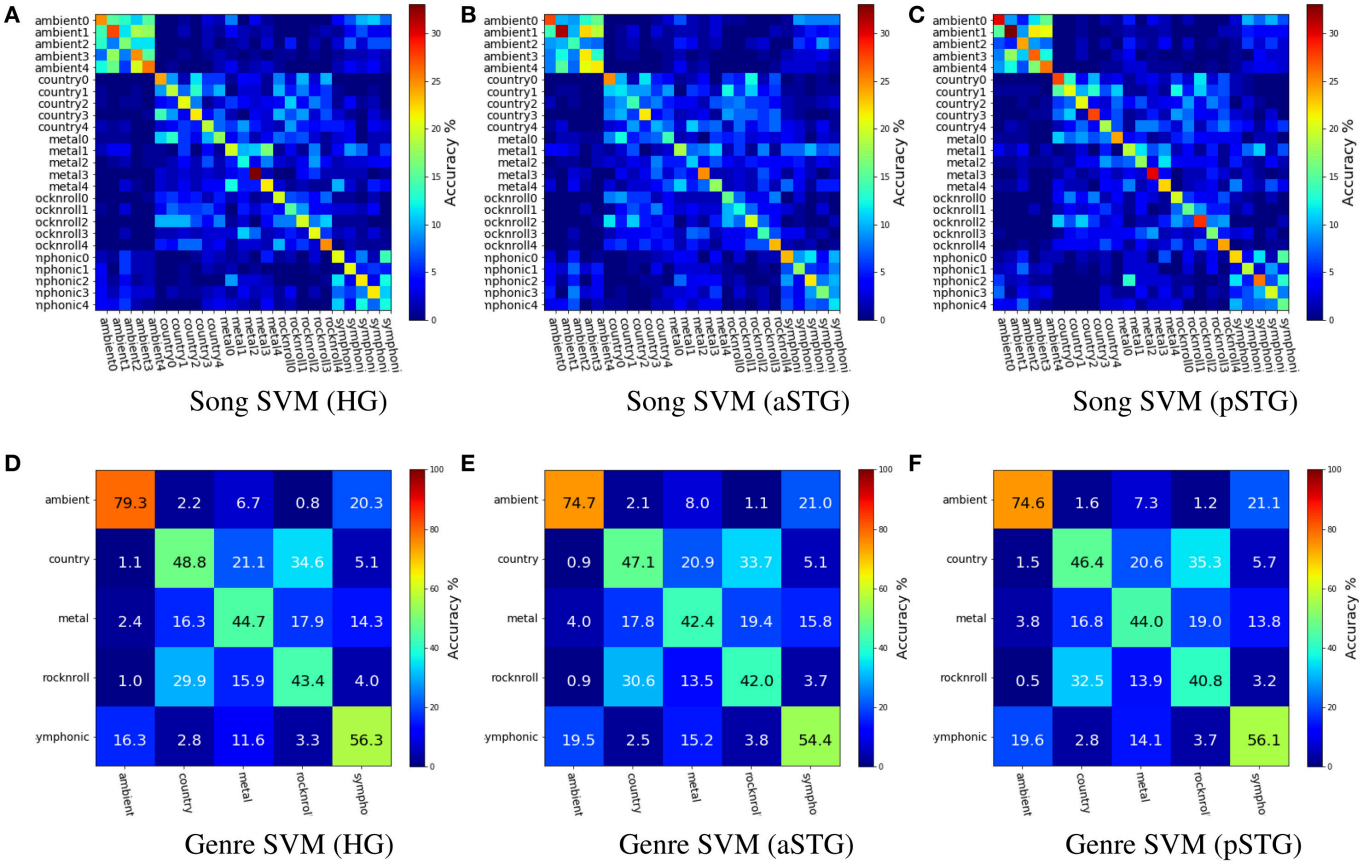
Group-level mean accuracies for song (upper) and genre (lower) SVM classifiers for voxels in Heschl's gyrus (HG), anterior superior temporal gyrus (aSTG), and posterior superior temporal gyrus (pSTG). The figures show that distinct anatomical ROIs yield similar relational information about music. The patterns of misclassifications show that when songs are misclassified, they are more likely to be confused with items from the same genre, or a similar sounding genre: e.g., Ambient and Symphonic; and Rock-n-Roll and Country. **(A)** Song SVM (HG). **(B)** Song SVM (aSTG). **(C)** Song SVM (pSTG). **(D)** Genre SVM (HG). **(E)** Genre SVM (aSTG). **(F)** Genre SVM (pSTG).

Figure 2 shows the group-averaged cross-validated results of within-subject SVM classification for the three bilateral temporal-region ROIs used for Analysis 1. Song classification results, with balanced cross-validation by run (Chance = 4%), were: HG (Mean = 21.1%, SE = 0.9%), aSTG (Mean = 18.5%, SE = 1.1%), and pSTG (Mean = 23.2%, SE = 1.0%). Results for 5-way genre classification, with balanced cross-validation by stimulus (Chance = 20%), were: HG (Mean = 54.5%, SE = 5.9%), aSTG (Mean = 52.1%, SE = 5.4%), and pSTG (Mean = 52.4%, SE = 5.5%).

**Figure 3:**
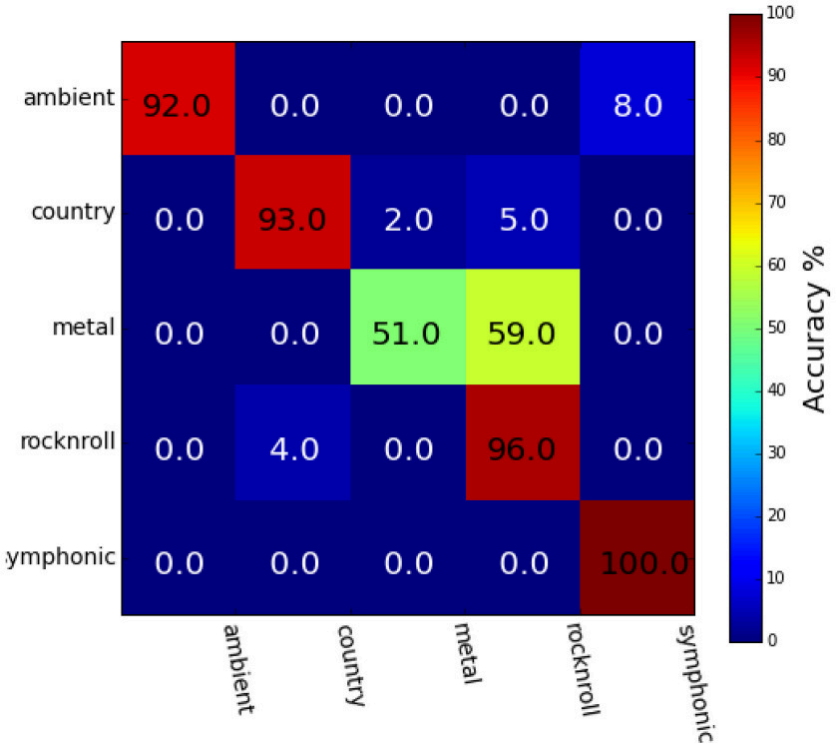
Group-averaged behavioral genre category-assignment confusions (*n* = 20; Mean = 86.4%, *SE* = 8.0%, Chance = 20%). Category confusions overlap those shown in Figure 2, with predominant confusions occurring between the Ambient and Symphonic genres, and also between the Country, Heavy Metal, and Rock&Roll genres.

Figure 3 shows the results of behavioral genre categorization (*n* = 20) for the 25 stimuli used in the genre classification task. Accuracies in the behavioral task (Mean = 86.4%, SE = 8.0%, Chance = 20%) were higher than the SVM classifier reported above. The Spearman rank-order correlation scores, *r*, between the group-averaged confusion matrix of the behavioral task and the group-averaged confusion matrix for the genre classifier for each ROI were HG (*r* = 0.76, *p* < 0.01), aSTG (*r* = 0.79, *p* < 0.01), and pSTG (*r* = 0.79, *p* < 0.01). The spearman rank-order correlation was calculated using the values above the main diagonal of the confusion matrices only, so as to remove positive correlation bias due to the diagonal structure of confusion matrices (Guntupalli, 2013).

### 3.2. Analysis 2

**Figure 4:**
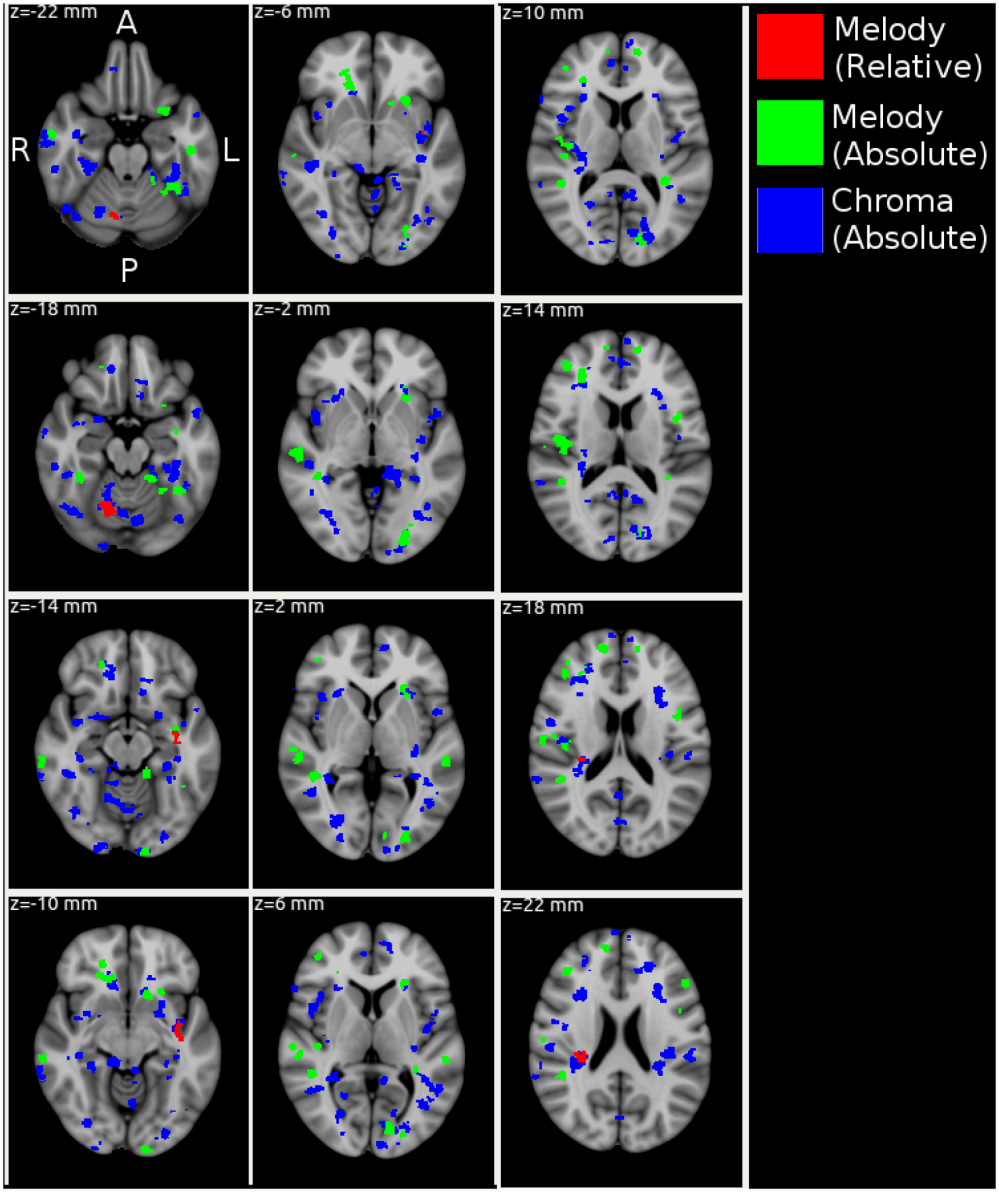
MNI-space group-level FWE-corrected clusters (*p* < 0.05) organized in 4 mm-spaced axial columns. In this multivariate analysis, the map value for each voxel derives from the information present in a 3-voxel-radius searchlight volume (max 123 voxels) and not each voxel individually. Acoustic chromagram features yielded the greatest number of significant clusters, 97 (43 left, 47 right, 7 both hemispheres), followed by absolute-pitch melody features (15 left, 12 right, 2 both), then relative-pitch melody features (1 left, 1 right, 1 both).

Figure 4 and Table 2 show MNI-space group-level FWE-corrected clusters (*p* < 0.05) based on stimulus-model-based encoding prediction accuracies (correlation-error probabilities). Significant clusters were identified for all three feature representations–melody relative pitch, melody absolute pitch, and acoustic chromagram features–in multiple sites spanning the searchlight regions of interest (ROIs). Acoustic chromagram features yielded the greatest number of significant clusters, 97 (43 left, 47 right, 7 both hemispheres), followed by absolute-pitch melody features (15 left, 12 right, 2 both), then relative-pitch melody features (1 left, 1 right, 1 both). Significant chromagram (Chrom) feature clusters occupied a total volume 10,595 voxels, spanning sites in most of the bilateral searchlight ROI volume: namely, temporal primary and secondary auditory cortex (A1, A2)–including Heschl's gyrus (HG), planum temporale (PT), superior temporal gyrus (STG), supramarginal gyrus (SMG), middle temporal gyrus (MTG) all lateralized marginally to the right hemisphere; Rolandic operculum (ROL); inferior frontal gyrus (IFG); temporal, frontal, and occipital poles (TP, FP, OP); middle frontal gyrus (MFG)/Broca's area; frontal orbital cortex (FO); intracalcarine cortex (CAL); insular cortex (IC); lingual gyrus (LING); parahippocampal gyrus (PHG); cerebellum; and multiple visual areas (V1, V2, V3, V4).

**Table 2:** Average group results: searchlight-based (sphere radius = 3 voxels, max. size 123 voxels) cross-validated within-subject stimulus encoding (*n* = 20; ridge regression).

					**Center of mass (MNI)**				
**#**	**Voxels**	**Max**	**Mean**	**Std**	***X***	***Y***	***Z***	***p*_clus._**	**Structure**
**Melody (Relative)**									
1	122	0.84	0.67	0.21	73.8	57.0	54.9	0.0110	Occipital Fusiform Gyrus
2	100	0.85	0.76	0.15	130.0	111.1	62.1	0.0174	Planum Polare
3	95	0.83	0.66	0.21	58.4	97.3	90.7	0.0174	Planum Temporale
**Melody (Absolute)**									
1	282	0.84	0.69	0.20	119.8	80.4	62.8	0.0001	Temporal Occip. Fusiform
2	281	0.86	0.78	0.13	58.5	95.9	86.9	0.0001	Planum Temporale
3	230	0.86	0.84	0.01	49.9	145.1	73.2	0.0001	Frontal Operculum Cortex
4	209	0.84	0.63	0.25	104.7	54.2	76.1	0.0001	Intracalcarine Cortex
5	206	0.83	0.69	0.21	68.7	53.5	75.7	0.0001	Intracalcarine Cortex
6	164	0.85	0.78	0.14	121.8	147.2	87.9	0.0004	Frontal Operculum Cortex
7	161	0.83	0.76	0.14	75.1	66.7	59.4	0.0004	Lingual Gyrus
8	136	0.84	0.69	0.21	54.4	144.0	95.2	0.0013	Inferior Frontal Gyrus
9	125	0.86	0.78	0.15	99.4	85.9	68.7	0.0019	Parahippocampal Gyrus
10	112	0.85	0.64	0.26	60.1	90.3	52.5	0.0036	Temporal Fusiform Cortex
11	101	0.82	0.65	0.23	147.1	90.5	99.8	0.0064	Parietal Operculum Cortex
12	90	0.86	0.77	0.18	52.8	111.1	80.7	0.0117	Insular Cortex
13	85	0.86	0.79	0.13	103.2	88.8	59.5	0.0149	Parahippocampal Gyrus
14	80	0.87	0.86	0.00	139.9	154.4	94.1	0.0191	Inferior Frontal Gyrus
15	78	0.85	0.81	0.07	134.7	131.8	93.0	0.0204	Precentral Gyrus
**Chroma (Absolute)**									
1	503	0.83	0.70	0.19	119.8	80.4	62.8	0.0001	Temporal Occip. Fusiform
2	422	0.83	0.68	0.22	58.5	95.9	86.9	0.0001	Planum Temporale
3	373	0.85	0.74	0.18	49.9	145.1	73.2	0.0001	Frontal Operculum Cortex
4	304	0.84	0.66	0.22	104.7	54.2	76.1	0.0001	Intracalcarine Cortex
5	280	0.84	0.67	0.23	68.7	53.5	75.7	0.0001	Intracalcarine Cortex
6	276	0.83	0.64	0.24	121.8	147.2	87.9	0.0001	Frontal Operculum Cortex
7	273	0.84	0.66	0.22	75.1	66.7	59.4	0.0001	Lingual Gyrus
8	270	0.83	0.69	0.18	54.4	144.0	95.2	0.0001	Inferior Frontal Gyrus
9	257	0.86	0.77	0.14	99.4	85.9	68.7	0.0001	Parahippocampal Gyrus
10	182	0.82	0.66	0.20	60.1	90.3	52.5	0.0002	Temporal Fusiform Cortex
11	181	0.85	0.67	0.23	147.1	90.5	99.8	0.0002	Parietal Operculum Cortex
12	167	0.84	0.56	0.28	52.8	111.1	80.7	0.0003	Insular Cortex
13	159	0.83	0.70	0.17	48.6	120.0	50.4	0.0003	No label found!
14	159	0.85	0.69	0.18	139.9	75.1	78.8	0.0003	Middle Temporal Gyrus
15	157	0.83	0.72	0.17	72.1	160.3	55.6	0.0003	Frontal Orbital Cortex

*The table lists statistics (size, max/mean/std accuracy) as well as localization information (coordinates in mm MNI152) for clusters with above-chance classification performance in the group (FWE-corrected, cluster-level probability p < 0.05)*.

Clusters due to absolute-pitch melody features occupied a total volume of 3,276 voxels and were concentrated in temporal and frontal areas largely overlapping those of chromagram features, but with fewer and smaller significant clusters. Notable differences in the distribution of clusters compared with chromagram features were the inclusion of clusters in the putamen; a greater presence of clusters in right MTG and STG; and left-lateralized clusters in multiple visual areas (V1, V2, V3, V4). Finally, relative-pitch melody features exhibited clusters that occupied a total volume of 317 voxels which were lateralized and concentrated in three clusters: the junction of the right cerebellum (c-VI) and temporal-occipital fusiform gyrus (FFG), left planum polare (PP), and right PT (A2). We observed overlapping representations of all three feature representations in the left PP. Outside of this area, relative-pitch and absolute-pitch melody features had no further overlapping clusters. Chromagram and relative-pitch melody clusters overlapped in the right cerebellum and in the right PT extending through the parietal operculum (PO) area of A1. Chromagram and absolute-pitch clusters overlapped in numerous sites that were mostly lateralized to the right: pMTG, HG, PT, FFG, CAL, FO, SMG, FP, IFG.

## 4. Discussion

### 4.1. Analysis 1

The within-subject song classification results show significantly higher accuracies than previously reported in Guntupalli (2013) (Mean = 15.95%, SE = 1.62%, Chance = 4%) for the same stimuli using different subjects with different (3T) fMRI data. One reason for the greater accuracies in the current study may be the use of high-field (7T) fMRI data, which doubles the spatial resolution of voxels in each dimension thus affording greater detail for pattern discrimination. Differences in voxel selection strategies are enumerated below.

The within-subject genre classification accuracies are slightly lower than those reported in Casey et al. (2012) (Mean = 60.0%) for the same stimuli, but with more stringent cross validation in the current study, and ≈25% lower than those reported in Guntupalli (2013) for the same stimuli. Apart from the use of high-field fMRI in the current study, differences between the current and the two former studies include 5,000-voxel feature selection by ROI in the current study, no sensitivity based selection in Casey et al. (2012), and 1,000-voxel feature-selection from whole brain voxels in Guntupalli (2013). The latter study also employed a different cross-validation scheme, which also accounts for some of the difference in accuracy. In the case of genre classification, selection of voxels from the whole brain 3T data yielded greater classifier accuracies than restricting voxel selection to temporal cortex with 7T data. Overall, these results show that distinct anatomical ROIs yield similar pattern-space information about song identity and genre, thus they hierarchically encode multiple levels of music information.

The high correlation score between behavioral and classifier confusion matrices is due to both exhibiting the same pattern of confusions between Ambient and Symphonic categories, and between Country and Rock&Roll categories. The most prominent difference between these two groups of confusions is that the confusion between Rock'n Roll and Heavy Metal in the behavioral task is much greater than it is with the classifier. These classification results show that songs that are misclassified at either the song level or the genre level are more likely to be confused with items from the same genre, or with items from a *similar-sounding* genre: e.g., Ambient and Symphonic, and Rock-n-Roll and Country. The latter implies that there is a super-ordinate category above the level of genre, one possibility for which is the presence or absence of vocals and/or percussion.

### 4.2. Analysis 2

In the realm of schematic feature representations, Janata et al. (2002) showed how dynamic attributes of tonal music, namely key changes, can be mapped onto a consistent cortical topography in prefrontal areas. Furthermore, they showed that the “tonality surface” representation was invariant to changes in the starting reference key, when the study was repeated with the same subjects over multiple scanning sessions. Hence, they demonstrated a direct cognitive representation of relative pitch encoding. In our work, we also found group-level representations of relative pitch, but for melodic encoding, rather than the slowly varying key surface of the previous work. Foster and Zatorre (2010) implicated IPS in the manipulation of auditory representations, such as used in a melodic transposition memory task. Whilst we found no significant clusters in the vicinity of IPS for relative-pitch melody features, we surmise that the naturalistic listening condition of the current study–i.e., attentive listening without an explicit memory task–elicited a differing view of voxel response patterns to relative pitch encoding of melodies than did the earlier work. Our relative-pitch results do however overlap with Janata et al. (2002) who also found in their tonality study with key that relative-pitch representations were present in the cerebellum and hippocampus, as well as in pre-frontal areas, both of which are present in our results.

Alluri et al. (2013) used an aggregate stimulus encoding model to perform voxel-wise response predictions to novel stimuli. Since the features were aggregated, they were not able to map responses to individual musical attributes. However, their aggregate model prediction results anatomically overlap with the current study, in that they found significant model-prediction accuracies in primary and secondary auditory areas (STG, HG, MTG), as well as pre-frontal and frontal areas (SFG), Rolandic operculum, putamen, and insula. In their earlier work, Casey et al. (2012) demonstrated stimulus-encoding-model-based decoding for low-level audio features corresponding to chromagram, spectral, and cepstral audio features. Chromagram features performed significantly above chance level in predicting the brain response for superior-temporal regions. In the current study, we have found wide activation of acoustic chromagram features across the all cortical and subcortical ROIs of the searchlight analysis. However, we note that the acoustic feature has folded within it the acoustic confounds described in Section 2, so components of the chromagram feature for acoustic mixtures, as in naturalistic music stimuli, may elicit sensitivities across many ROIs because the feature encodes substantial additional information beyond the intended representation of polyphonic pitch content of the stimulus.

## 5. Conclusion and future directions

We have demonstrated parallel, distributed, and overlapping representations of musical features using machine learning models, high-field fMRI, and naturalistic music stimuli. The results from Analysis 1 show that decoding models can identify songs significantly above chance levels by their voxel pattern responses for held-out runs, and that categorical models accurately decode music genre categories for voxel pattern responses to novel stimuli in five genres. Furthermore, the pattern of confusions exhibited by the classifiers was significantly correlated with confusions in a behavioral categorization task. These results support our hypothesis that music cognition is neurally represented by multivoxel pattern spaces whose geometric properties, such as distance between response vectors, underlie observed human musical behavior.

Results from Analysis 2 demonstrate that stimulus-model-based-encoding accurately predicts voxel responses to music stimuli yielding significant clusters in multiple sites across the cortical volume. As we expected to see, distinct musical features are differentially encoded in distributed and anatomically overlapping sites. The current study extends prior work in stimulus-model-based encoding of music representational spaces by providing maps, not only of audio-based feature encoding, but also of schematic music features. Mapping parallel features of the information content in music content reveals wide networks of overlapping representational spaces for music. Future work will explore how well different pitch and rhythm representational space hypotheses, such as the tonnetz and the simplex models, can predict multivoxel responses in areas known to be implicated in the processing of these musical attributes, which will allow us to select the most likely neurally encoded representation among competing representational hypotheses for specific musical attributes.

We note, however, that care must be taken when extracting acoustic features to avoid confounding within the feature multiple unintended attributes of the stimulus along with the intended musical attribute, as we observed with the chromagram feature. This highlights a potentially important advantage of symbolic music features for mapping music cognition, and it also throws into question the utility of mixed low-level audio features for mapping music representations across cortical volumes. Audio source separation methods, which are the subject of much current music informatics research, may proove useful for increasing the representaitonal specificity of automatic acoustic feature extraction.

Our future work will include regrouping our analyses, separating results by genre, to test the hypothesis that music is cortically organized by high-level categories–such as genre, emotion, and semantic categories–with lower-level schematic and acoustic features repeatedly embedded within these superordinate representational spaces. The current study modeled stimulus-synchronous imaging. A further refinement to our work would be to introduce of models for predictive stimulus encoding, in which features of current and past time steps predict future voxel responses. Such models would be necessary to illuminate the neural representation of prediction-driven mechanisms that are widely understood to be implicated in the anticipation and reward mechanisms of musical enjoyment.

## Author contributions

MC designed the experiments, prepared the stimuli, prepared data and software for analysis of the 3T and 7T images, analyzed the data to produce the results in this paper, and wrote the paper.

### Conflict of interest statement

The author declares that the research was conducted in the absence of any commercial or financial relationships that could be construed as a potential conflict of interest.
